# A New “Dispensary”

**Published:** 1862-03

**Authors:** 


**A NEW "DISPENSARY."**—To "poor white folks," and folks of every color, caste, and condition; to strangers who may be in New-York on any Sunday, who have no acquaintances, no friends, no money, nor any thing; to those who are kept here, expecting a remittance, which comes never; to all such who would like to "go to meeting," without the chance of having to stand half an hour, waiting for some slow or impertinent sexton to show him a seat up in the gallery, or under the gallery, on hard boards and without books; or that other chance, if placed in an eligible pew, of being invited out two or three times in the course of the service; or the still other chance of walking a mile or two up-town, on a scorching day, in search of an open church, without finding one, as if there were no religion in "dog-days," and the "old boy" had ceased hostilities for a spell, or was "out of town;" to all such it may be comforting to know that an elegant house of religious worship has been erected opposite the Fourteenth street front of the Academy of Music, where the Gospel is preached "without money and without price;" absolutely free to all; every pew-door is hewn away; and any unoccupied seat is as much yours as any millionaire's in the land; nobody has a right to bow you out of it. Services are held at half-past ten o'clock on Sunday mornings, and at half-past seven, Sunday and Wednesday nights, the year round; also on Sunday afternoons, at about four in summer, and three in winter. Every pew is plentifully supplied with books, in large print and small, with cushioned seats, and stools, and carpeted floor; it is the coolest, best lighted, and best ventilated church we have seen in this city, for its size and object. The minister neither whispers nor lisps, nor mumbles his words as if his mouth were filled with hot mush; nor are his tones so sleepy or so terribly solemn as to keep you in mind of a dungeon or a graveyard; nor is he a half-fledged divinity-student, practicing on the poor; nor is he an infirm or "weak brother," just put in to fill up the place; nor is he so painfully meek as to be unable to charge your sins upon you, or to stumble at such words as "hell" and "damnation;" but he is an educated, earnest, fearless, practical preacher; a man who does his work with a will; he speaks as by "authority," and not "by your leave, sir." Blessing and honor be to the hearts which devised, and to the purses that caused to be executed, the enterprise on Fourteenth street, near Irving Place. Now let some of our rich city subscribers complete the work, by founding a sextonship and a pastorship; and let their names be graven on the portals of the church, to be lisped with grateful memories by the poor, the friendless, and the stranger, for all time to come. And thus will the great and liberal city of New-York, while she has a number of medical "Dispensaries," where the penniless can have all the medicines they need for themselves and families, merely for the asking, may have at least one spiritual dispensary, where remedies for a mind diseased are administered absolutely free; where the struggling children of poverty may be fed with "the bread which cometh down from heaven," and drink from that fountain opened in the side of Him who bled on Calvary; where there will be found "a balm for every wound, a cordial for every fear;" and where the forsaken and the despondent, the weary and the struggling, may find and feel that there is one to be heard of who "sticketh closer than a brother," and that on application to Him for relief, adapted to their various situations, they "shall in no wise be cast out."

